# Reply to the opinion paper “The EU chemicals strategy for sustainability: an opportunity to develop new approaches for hazard assessment” by Scholz et al.

**DOI:** 10.1007/s00204-022-03319-w

**Published:** 2022-06-10

**Authors:** Matthias Herzler, Philip Marx-Stoelting, Ralph Pirow, Christian Riebeling, Andreas Luch, Tewes Tralau, Tanja Schwerdtle, Andreas Hensel

**Affiliations:** German Federal Institute for Risk Assessment (BfR), Max-Dohrn-Str. 8–10, 10589 Berlin, Germany

## Introduction

We thank the authors of the above opinion paper (Scholz et al. 2022) for their contribution to the ongoing discussion of the European Commission’s “Chemicals Strategy for Sustainability” (CSS; European Commission 2020), in which they take a critical look at our commentary published in this journal in June 2021 (Herzler et al. 2021). After all, our most important goal with that commentary was to stimulate a scientific debate of the CSS, a debate the extent of which we still find in need of improvement, given the strategy’s potentially massive impact on the system of chemicals regulation as well as society as a whole.

We feel that the Scholz et al. commentary falls somewhat short of the expectations its title raises, in particular in demonstrating where the CSS compared to the current regulatory landscape provides increased opportunities for new hazard assessment approaches and also in explaining why it should be advantageous to focus on hazard, not risk assessment methodologies. However, our general impression is that altogether our views may in fact not be as far apart as the authors seem to suggest. Discrepancies, where noted, can in many cases be explained by the different perspective and responsibilities of academic research institutions compared to a government authority. In other parts, the line of argumentation used by the authors follows a reasoning popular in environmental risk assessment but maybe less so in the world of human health assessment. In some areas, finally, apparent differences in opinion arise from the fact that the positions we took in our comment of June 2021 are not reflected in a fully accurate way.

Below, we would like to briefly expand on these issues following the structure provided by Scholz and colleagues.

## Toxic-free environment

In our comment, we remarked that the terminology used in the CSS is often imprecise and unclear, e.g., regarding terms such as “pollution” or “toxic-free”. We further pointed out that, if the latter was understood in the same way as the definition of “non-toxic environment” coined by the European Commission in 2017  (European Commission 2017), i.e., “…an environment that is free of pollution [sic] and of exposures to hazardous chemicals at levels that are harmful to human health and to the environment…”, then such a goal would only reflect the already existing paradigm of the current system of chemicals regulation in the EU, i.e., the principle that chemicals should only be placed on the market if their safe use is ascertained.

Scholz et al. seem to refer to this definition of “non-toxic” or “toxic-free” environment and, hence, there is no disagreement between us that this should indeed be the goal of chemicals regulation. We would like to point out, however, that the European Commission appears to follow a much more rigid interpretation according to which hazardous chemicals should be removed from the environment, regardless of whether they actually occur at harmful levels or not. Contrary to the perception of Scholz et al., i.e., that the CSS was just a “political mandate” or “policy statement”, purely hazard-based regulation concepts, such as the generic approach to risk management (GRA), are currently being implemented at record-breaking speed, leaving little-to-no room for further scientific discussion.

## The precautionary principle

Scholz and colleagues also refer to the precautionary principle. In our commentary, we highlighted some of the pre-requisites for its application according to the Commission’s own communication of 2000 (European Commission 2000), i.e., that measures taken under this principle should be “proportional to the chosen level of protection, non-discriminatory in their application, consistent with similar measures already taken, based on an examination of the potential benefits and costs of action or lack of action (including, where appropriate and feasible, an economic cost/benefit analysis), subject to review, in the light of new scientific data, and capable of assigning responsibility for producing the scientific evidence necessary for a more comprehensive risk assessment.”

These requirements represent principles of good regulatory practice, which every EU citizen can expect to be followed by the authorities. In this regard, chemicals regulation does not differ (much) from tax or traffic regulation and the BfR, as a public authority with a strong legal mandate to provide policy advice, is bound by this principle.

However, there is a fundamental problem with the proportionality of a purely hazard-based regulation: due to the very nature of such a regulation, proportionality cannot be established. If the risk addressed by the regulatory measure (or, expressed positively: the health/environmental benefit achieved by it) cannot be quantified at least to some extent, then no cost–benefit analysis can be carried out. Moreover, if also the societal costs of the measure are not evaluated, there is then practically no basis for deciding whether a planned measure is proportional or even justified.

It is therefore a valid (and actually commonly applied) approach, as Scholz et al. remark, to use a hazard-based assessment only as a first indicative tier which then triggers a further, more refined characterisation of the potential problem. It could also be appropriate to implement measures based on hazard with demonstrated high benefit, if they come at little cost to society. However, as pointed out above, this is not what is currently planned in the context of the implementation of the CSS.

## Evidence for sufficient protection of human health regarding chemical exposure by existing regulation

Our commentary specifically analysed (and questioned) the evidence brought forward by the Commission in support of their claim that the current system of chemicals regulation exposes EU citizens to significant health risks, and that therefore urgent and rigorous action was required “to restore human health to a good status”.

In particular, we showed that even very strong claims made in the CSS such as “combined prenatal exposure to several chemicals has led to reduced foetal growth and lower birth rates” were only insufficiently substantiated in the CSS itself or the associated staff working documents (SWDs). Since specific evidence was not provided, we therefore looked at generic health indicators for the EU population and concluded that also these figures did not positively suggest health problems of a dimension that would seem to require immediate action. However, we certainly never concluded that they proved the absence of any relevant chemical risk, which would obviously constitute a logical fallacy. We also never claimed that they gave proof of the success of chemical risk assessment (although we would obviously like to hope so), only that they did not lend support to the opposite view. In addition, in our commentary, we did not discuss (nor did we deny) chemical-related health problems on a global scale. Our reflections focused on the situation in the EU with its comprehensive system of chemicals regulation, because this is the correct reference point for discussing possible benefits or drawbacks of the measures intended to be implemented under the CSS.

In summary, and in contrast to the respective statement by Scholz et al., we did not deny the necessity of additional measures to ensure chemical safety for the EU citizens in principle. On the contrary, we explicitly highlighted the constant need of the regulatory system for adaptation to scientific and technical progress. Our criticism, however, was directed towards the alleged need for urgency by means of which the Commission empowered itself to start the implementation of far-reaching regulatory measures with only little involvement of the scientific community and with only rudimentary impact assessment.

The argument of urgency is in fact central to the CSS implementation plan. Without it, there would be enough time to wait for current and planned research projects to deliver new insights pertinent to identifying real problems in need of regulation and to finding the best, most efficient solutions for targeting them. In their opinion paper, Scholz et al. express their hope for “an opportunity to revise, modernise, and improve current hazard and risk assessment procedures based on sound science”.

Already in previous years, BfR has addressed this issue in a number of completed projects contributing constructive, scientific proposals to address complex issues such as mixture toxicity (Tralau et al. 2021) or engaging in the promotion of new approach methodologies (NAMs) (Escher et al. 2019; Moné et al. 2020; Rovida et al. 2020, 2021). Currently, we are active in a number of complex EU research projects with exactly this goal, e.g., with respect to the implementation of New Approach Methodologies and Next Generation Risk Assessment (https://www.risk-hunt3r.eu/), mixture toxicity (https://panoramix-h2020.eu/, together with UFZ), and will strongly engage in the partnership for the assessment of risk from chemicals (PARC, https://www.anses.fr/en/content/european-partnership-assessment-risks-chemicals-parc). However, such projects take time, which, according to the CSS, is not available—because of the alleged urgency.

Another argument repeatedly brought forward by Scholz et al. refers to the high number of chemicals to which humans are exposed today, as an argument indicating—in their view—the need for action. This, on its own, appears debatable: the human body contains countless chemicals (requiring them for its survival) and is exposed to countless more from the natural environment (some actually quite critical from the perspective of risk assessment). Another classical example, often overlooked, is food. Toxicologically speaking, eating means no less than the deliberate consumption of complex mixtures consisting of thousands of chemical food constituents day after day. Moreover, many of these substances will be perfectly bioavailable once taken up, with only a fraction being “non-hazardous” (think of sugar, salt, ethanol, glycyrrhizic acid, phytoestrogens, hormones in meat, etc.).

In fact, one of the major functions of the body is to secure its physiological and biochemical homeostasis in an ever-changing chemical environment. Identifying a particular chemical, e.g., in human urine could point at its previous presence in the blood at critical levels, or it could merely be a sign of the body “doing its job” of eliminating a chemical it has no use for. While our understanding of the meaning of biomonitoring results has certainly increased over the last years, there is still much to learn, in particular with respect to quantitative aspects, and it seems inappropriate to conclude on a health risk from the mere presence of a chemical in body fluids.

The same holds for mixture risks. Possible additive action of chemicals sharing the same or interconnected modes of action is an established scientific fact, as pointed out in our commentary to the CSS, in which we also listed further requirements to be fulfilled to create a real-life problem. With regard to human health, these boundary conditions for mixtures to become a toxicologically relevant problem are scientifically well defined as is the potential chemical space for which this might indeed be of regulatory relevance. The available data indicate that this space is confined to a limited number of substances that could become relevant in regulatory terms only in scenarios where single substance-based assessment of the main mixture risk drivers leaves a significant regulatory gap (Herzler et al. 2021; Tralau et al. 2021). To some extent, mixture toxicity also has been in the focus of “classical” and regulatory toxicology already, particularly at the BfR. Moreover, mixture hazard assessment concepts are already well established, whereas the main scientific challenge, in our view, will be to direct regulatory resources to those scenarios of real-life concern, for which scientifically sound, risk-based assessment and regulation concepts need to be developed.

Last, but not least, in the Scholz et al. commentary, we missed a balanced discussion taking account of how man-made chemicals are also the foundation of human civilisation on earth. Humans need them for their health and safety, as well as for the free development of their personalities. On a societal scale, there will be no coping with the challenges of climate change without man-made chemicals. Of course, considering human and environmental health aspects already at the product development stage, to produce “safe-and-sustainable-by-design” chemicals, can only be supported. But where this is not feasible, industrial production will continue to require reactive (and therefore often also bioreactive) chemicals. In such cases, care must be taken to fully understand the consequences of broad and blunt regulatory measures before their implementation. As pointed out above, purely hazard-based regulation does not seem well equipped in this regard.

## Summary

In conclusion, we would like to underscore again that we are not in denial of the constant need of adapting chemical risk assessment methodology to the scientific state of the art. In fact, this was already prominently highlighted in our commentary of June 2021. Moreover, we are actively advocating for a strong role of science in chemicals regulation. We agree with Scholz et al. in that the CSS has highlighted some of the most pressing questions in our line of work. As scientists, we should now make sure that the best science is used rather than trying to bypass it by unsubstantiated shortcuts.
